# Improvement of early miscarriage rates in women with adenomyosis via oxytocin receptor antagonist during frozen embryo transfer-a propensity score-matched study

**DOI:** 10.1186/s12958-024-01255-1

**Published:** 2024-07-12

**Authors:** Po-Wen Lin, Chyi-Uei Chern, Chia-Jung Li, Pei-Hsuan Lin, Kuan-Hao Tsui, Li-Te Lin

**Affiliations:** 1 Department of Obstetrics and Gynecology, Kaohsiung Veterans General Hospital, No.386, Dazhong 1st Rd., Kaohsiung City, Zuoying Dist 81362 Taiwan; 2 Department of Nursing, Shu-Zen Junior College of Medicine and Management, Kaohsiung City, Taiwan; 3 School of Medicine, College of Medicine, National Sun Yat-sen University, Kaohsiung City, Taiwan; 4 Department of Biological Science, National Sun Yat-sen University, Kaohsiung City, Taiwan; 5 Institute of Biopharmaceutical Sciences, National Sun Yat-sen University, Kaohsiung City, Taiwan; 6 Institute of Medicine, Chung Shan Medical University, Taichung City, Taiwan

**Keywords:** Oxytocin receptor antagonist, Atosiban, Adenomyosis, Frozen embryo transfer, In vitro fertilization

## Abstract

**Background:**

Dysfunctional uterine peristalsis seems to play a pivotal role in hindering embryo implantation among women diagnosed with adenomyosis. This research aims to investigate whether administering an oxytocin receptor antagonist during a frozen embryo transfer (FET) cycle using a hormone replacement therapy (HRT) protocol can enhance in vitro fertilization (IVF) outcomes for infertile women affected by adenomyosis.

**Methods:**

Between January 2018 and June 2022, our reproductive center conducted IVF-FET HRT cycles for infertile women diagnosed with adenomyosis. Propensity score matching was employed to select matched subjects between the two groups in a 1:1 ratio. Following this, 168 women received an oxytocin receptor antagonist during FET, constituting the study group, while the matched 168 women underwent FET without this antagonist, forming the control group. We conducted comparative analyses of baseline and cycle characteristics between the two groups, along with additional subgroup analyses.

**Results:**

The study group exhibited notably lower rates of early miscarriage compared to the control group, although there were no significant differences in clinical pregnancy rates, ongoing pregnancy rates, and live birth rates between the two groups. Multivariate analysis revealed a negative correlation between the use of oxytocin receptor antagonists and early miscarriage rates in women with adenomyosis. Subgroup analyses, categorized by age, infertility types, and embryo transfer day, showed a substantial decrease in early miscarriage rates within specific subgroups: women aged ≥ 37 years, those with secondary infertility, and individuals undergoing day 3 embryo transfers in the study group compared to the control group. Furthermore, subgroup analysis based on adenomyosis types indicated significantly higher clinical pregnancy rates, ongoing pregnancy rates and live birth rates in the study group compared to the control group among women with diffuse adenomyosis.

**Conclusions:**

Administering an oxytocin receptor antagonist during FET may reduce the early miscarriage rates in women with adenomyosis.

**Supplementary Information:**

The online version contains supplementary material available at 10.1186/s12958-024-01255-1.

## Introduction

Uterine dynamics involve the rhythmic contraction known as uterine peristalsis, which generates endometrial waves throughout the menstrual cycle. During the luteal phase, the uterus typically maintains a calm state favorable for embryo implantation [1, 2]. Embryo transfer (ET) marks the critical final step of in vitro fertilization (IVF). Studies have shown that increased uterine contractile activity during ET in IVF cycles is linked to decreased chances of successful pregnancies [3–5]. A meta-analysis revealed a notable negative impact of heightened contraction frequency (> 3 contractions/minute) on pregnancy rates [1]. In a prospective cohort study, the non-pregnant group consistently exhibited a higher frequency of uterine contractions compared to the pregnant group [6]. Hence, heightened uterine contractions during embryo implantation significantly reduce pregnancy rates, with higher frequencies of contractions exacerbating this adverse effect.

Oxytocin triggers contractions by binding to oxytocin receptors, primarily boosting intracellular calcium and prostaglandin levels. This action results in contractions of the uterine myometrium, ultimately leading to labor and delivery [7, 8]. Oxytocin receptor antagonists, which mimic oxytocin but with a stronger receptor affinity, work by decreasing prostaglandin production and calcium influx into cells, thereby inhibiting uterine contractions [9, 10]. Although oxytocin receptor antagonists are commonly used for tocolysis [11, 12], their application in ET is currently under investigation. Some studies have demonstrated a significant increase in clinical pregnancy rates with the use of atosiban during ET compared to controls [13–17], while others have not observed such benefits [18–21]. Randomized, double-blinded, controlled trials suggest that atosiban treatment during ET may not improve IVF outcomes in infertile women or those with recurrent implantation failure [22–24]. However, few studies have specifically focused on women with adenomyosis, a condition characterized by abnormal uterine peristalsis [25, 26].

Adenomyosis, characterized by the infiltration of endometrial glands and stroma into the myometrium, has been associated with adverse effects on IVF outcomes [27–29]. Dysfunctional uterine peristalsis appears to be a critical factor contributing to impaired embryo implantation in women with adenomyosis [1, 30, 31]. Therefore, we hypothesize that utilizing oxytocin receptor antagonists during ET could potentially enhance IVF outcomes in this population. However, there is a paucity of published research on this subject. Hence, our study aims to explore the effects of oxytocin receptor antagonist administration during frozen embryo transfer (FET) on IVF outcomes among infertile women with adenomyosis.

## Materials and methods

### Study design and participants

**Fig. 1:**
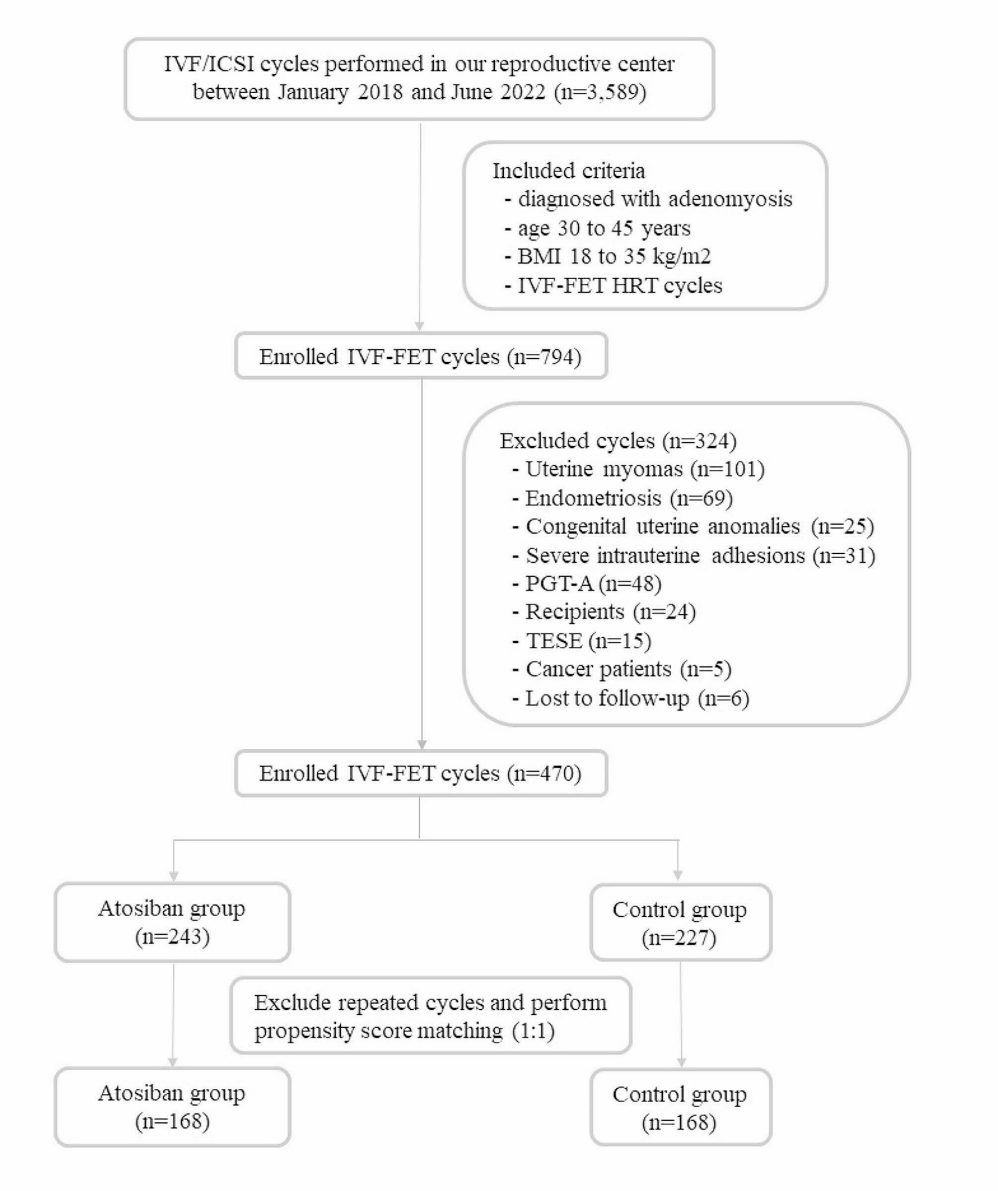
Study flowchart IVF, in vitro fertilization; ICSI, intracytoplasmic sperm injection; BMI, body mass index; FET, frozen embryo transfer; HRT, hormonal replacement therapy; PGT-A, preimplantation genetic testing for aneuploidy; TESE, testicular sperm extraction

The retrospective cohort study took place at the Reproductive Medical Center of Kaohsiung Veterans General Hospital from January 2018 to June 2022. Approval for the study was granted by the Institutional Review Board at Kaohsiung Veterans General Hospital (reference number: KSVGH23-CT5-06). Due to its retrospective nature, the need for consent was waived by the Institutional Review Board. Patient data were collected from electronic medical records and IVF treatment sheets during the specified period. The study enrolled infertile women diagnosed with adenomyosis who underwent IVF-FET cycles using a hormone replacement therapy (HRT) protocol at our reproductive center. Inclusion criteria specified patients aged 30 to 45 years with a body mass index (BMI) ranging from 18 to 35 kg/m^2^. Adenomyosis diagnosis was established via sonography conducted by a certified sonographer and subsequently confirmed by a physician based on the Morphological Uterus Sonographic Assessment (MUSA) criteria [32]. Ultrasound assessments were performed utilizing a Voluson E8 device (GE Healthcare, Chicago, U.S.A.) equipped with a transvaginal probe. Adenomyosis was further categorized as focal, diffuse, or adenomyoma, as described in prior literature [32–34]. Exclusion criteria included patients with uterine myomas, laparoscopic or sonographic evidence of endometrioma or pelvic endometriosis, congenital uterine anomalies, severe intrauterine adhesions, individuals undergoing preimplantation genetic testing cycles for aneuploidy (PGT-A), recipients of donated oocytes, husbands of patients undergoing testicular sperm extraction, cancer patients, and those lost to follow-up. A total of 470 cycles were included and allocated into either the study or control groups. The study group received an oxytocin receptor antagonist during the FET procedure, while the control group did not. The decision to administer the antagonist was based on individual patient consultation and preference following thorough discussions with a physician. If multiple cycles were performed by the same patient within the study period, repeated cycles were excluded. To reduce selection bias, propensity score matching (PSM) was employed to select matched subjects with balanced age, BMI, anti-mullerian hormone (AMH) levels, and endometrial thickness between the two groups in a 1:1 ratio. Following matching, 168 cycles in the study group and 168 cycles in the control group were analyzed. The study flowchart is presented in Fig. 1.

### Frozen embryo transfer protocol

All participants in this study underwent FET cycles following the HRT protocol with prior treatment using gonadotropin-releasing hormone (GnRH) agonist. A single subcutaneous injection of 3.75 mg long-acting GnRH agonist (Leuplin Depot, Takeda Pharmaceutical Company Limited, Yamaguchi, Japan) was administered on either Day 2 or 3 of the menstrual cycle. After 28 days, upon confirming a thin endometrium (< 5 mm) via transvaginal sonography, endometrial preparation began with daily oral estradiol doses ranging from 6 to 8 mg (Ediol, Synmosa Biopharma Corporation, Hsinchu County, Taiwan) alongside estradiol gel (Oestrogel gel, Besins, Drogenbos, Belgium). Following 14 days of consecutive administration, endometrial thickness was assessed through transvaginal ultrasound. Upon achieving an endometrial thickness of at least 8 mm, luteal phase support was initiated. This involved daily intravaginal application of 90 mg gel (Crinone 8% gel, Merck Serono, Hertfordshire, UK), daily oral intake of 30 mg dydrogesterone (Duphaston, Abbott, Olst, the Netherlands), and weekly intramuscular injections of 125 mg progesterone (Progeston Depot, Tafong Pharmaceutical Co., Ltd., Changhua City, Taiwan).

Our reproductive medical center adopted a freeze-all strategy for infertile women diagnosed with adenomyosis, wherein all embryos underwent cryopreservation using the vitrification technique. Thawing and transfer of cleavage-stage embryos occurred on the 4th day after progesterone administration, while blastocysts were transferred on the 6th day. Day 3 embryo quality assessment followed the criteria established by the Istanbul consensus workshop, evaluating cell number, blastomere symmetry, fragmentation percentage, and the presence of multinucleation. The optimal number of cells in a day 3 embryo is typically between 6 and 10. Ideally, the cells should be of equal size, as significant asymmetry can indicate poor embryo quality. Fragmentation, the presence of anucleate cytoplasmic fragments within the embryo, is graded as follows: Grade 1: No fragmentation or very few fragments (< 10%); Grade 2: Moderate fragmentation (10–25%); Grade 3: Significant fragmentation (25–50%); Grade 4: Severe fragmentation (> 50%). Each cell should ideally have only one nucleus, as multinucleation is associated with chromosomal abnormalities and lower implantation potential. The embryo’s morphology is graded as follows: Grade A (Excellent): 6–10 cells, symmetric, < 10% fragmentation, no multinucleation; Grade B (Good): 6–10 cells, slight asymmetry, 10–25% fragmentation, no multinucleation; Grade C (Fair): <6 or > 10 cells, moderate asymmetry, 25–50% fragmentation, possible multinucleation; Grade D (Poor): <6 or > 10 cells, severe asymmetry, > 50% fragmentation, frequent multinucleation [35]. In this study, top-quality Day 3 embryos were defined as Grade A (6–10 cells, symmetric blastomeres, < 10% fragmentation, and absence of multinucleation). Assessment of Day 5 embryo quality utilized the Gardner and Schoolcraft scoring system, considering the degree of blastocyst expansion, inner cell mass morphology and trophectoderm morphology. The degree of blastocyst expansion is graded on a scale from 1 to 6: 1: Early blastocyst, where the blastocoel (fluid-filled cavity) is less than half the volume of the embryo; 2: Blastocoel is more than half the volume of the embryo; 3: Full blastocyst, where the blastocoel completely fills the embryo; 4: Expanded blastocyst, with a blastocoel volume larger than the early embryo and a thinning zona pellucida; 5: Hatching blastocyst, where the trophectoderm is starting to herniate through the zona pellucida; 6: Hatched blastocyst, where the blastocyst has completely escaped from the zona pellucida. The quality of the inner cell mass is graded as follows: A: Tightly packed cells, many cells present; B: Loosely grouped cells, several cells present; C: Few cells, appearing irregular or disorganized. The quality of the trophectoderm is graded as follows: A: Many cells forming a cohesive layer; B: Few cells forming a loose epithelium; C: Very few large cells. Top-quality Day 5 embryos were identified as Grade 3AA at least. Embryo transfer was performed under transabdominal ultrasound guidance. In the study group, intravenous administration of atosiban (Tractocile 7.5 mg/ml, Ferring, Kiel, Germany) comprised a 6.75 mg bolus before transfer, followed by continuous infusion of the remaining 30.75 mg in 100 mL of normal saline at a rate of 15.4 mg/h, totaling 37.5 mg over 2 h. Progesterone supplementation was prescribed for both groups until 10–12 weeks of gestation upon pregnancy confirmation.

### Outcome measures

The primary outcome measure of this study was the live birth rates, defined as the delivery of a viable fetus beyond 24 weeks of gestation. Secondary outcomes included miscarriage rates, clinical pregnancy rates, and ongoing pregnancy rates. Miscarriage was defined as a loss of pregnancy occurring after visualization of fetal cardiac activity and before 24 weeks of gestation, with further subcategorization into early miscarriage (≤ 12 weeks of gestation) and late miscarriage (> 12 weeks of gestation). Clinical pregnancy was determined by the detection of fetal heartbeat at 6–7 weeks of gestation via transvaginal sonography, while ongoing pregnancy referred to pregnancies beyond 12 weeks of gestation.

### Statistical analysis

Nearest neighbor matching with a caliper of 0.02 was applied to calculate the PSM for baseline parameters in a 1:1 ratio for the two groups. The normal distribution of continuous variables was assessed using the Kolmogorov–Smirnov test. Independent t-tests were employed for quantitative variables, while categorical variables were analyzed using the Chi-square test. Multivariable logistic regression was conducted to determine independent effects, adjusting for covariates including age, BMI, duration of infertility, previous IVF attempts, types of infertility, AMH levels, endometrial thickness, day of ET, and the proportion of transferred embryos. Results are presented as odds ratios (ORs) with corresponding 95% confidence intervals (CIs). Statistical analyses were carried out using Statistical Package for Social Sciences (SPSS) version 20.0 (Chicago, IL, USA), with statistical significance set at *P* < 0.05.

## Results

Table 1 presents a comparison of baseline and cycle characteristics among the study population following propensity score matching. No significant differences were observed between the study and control groups in terms of age, BMI, duration of infertility, previous IVF attempts, types of infertility, causes of infertility, follicular stimulating hormone (FSH) levels, AMH levels. Additionally, endometrial thickness, day of ET, proportion of transferred embryos and proportion of at least one top-quality embryos transferred were similar between the two groups. Clinical pregnancy rates, ongoing pregnancy rates, and live birth rates were also compatible between the two groups. However, the study group exhibited a significantly lower rate of early miscarriage compared to the control group (8.1% vs. 21.5%, *P* = 0.015).

**Table 1:** Baseline and cycle characteristics of patients with adenomyosis undergoing either atosiban administration or no administration during frozen embryo transfer

Parameters	Atosiban (*n* = 168)	Control (*n* = 168)	*p* value
Age (years)	37.9 ± 4.2	38.2 ± 3.8	0.531
Body mass index (kg/m^2^)	23.7 ± 3.9	23.7 ± 4.3	0.970
Infertility duration (years)	4.7 ± 3.2	5.3 ± 3.7	0.102
Previous IVF attempts (n)	2.9 ± 2.5	3.1 ± 2.3	0.296
Types of infertility (%)			0.913
Primary infertility	47.0%(79/168)	47.6%(80/168)	
Secondary infertility	53.0%(89/168)	50.3%(88/168)	
Causes of infertility			0.817
PCOS	13.7%(23/168)	9.5%(16/168)	
Tubal factors	8.9%(15/168)	10.1%(17/168)	
Male factors	8.9%(15/168)	11.3%(19/168)	
DOR	22.6%(38/168)	25.0%(42/168)	
Unexplained	13.7%(23/168)	14.3%(24/168)	
Multiple	32.1%(54/168)	29.8%(50/168)	
FSH (IU/L)	5.5 ± 2.9	5.1 ± 2.9	0.163
Anti-Müllerian hormone (ng/mL)	3.36 ± 3.47	3.37 ± 2.87	0.963
Endometrial thickness (mm)	11.7 ± 2.3	11.6 ± 2.2	0.884
ET day (%)			0.258
Day 3 ET	60.1% (101/168)	66.1% (111/168)	
Day 5 ET	39.9% (67/168)	33.9% (57/168)	
Number of transferred embryos (%)			0.308
1 embryo	13.7% (23/168)	11.9% (20/168)	
2 embryos	58.3% (98/168)	50.6% (85/168)	
3 embryos	17.9% (30/168)	22.6% (38/168)	
4 embryos	10.1% (17/168)	14.9% (25/168)	
At least one top-quality embryos transferred (%)	79.2% (133/168)	78.6% (132/168)	0.894
Biochemical pregnancy rate (%)	58.9% (99/168)	48.2% (81/168)	0.049
Clinical pregnancy rate (%)	51.2% (86/168)	47.0% (79/168)	0.445
Ongoing pregnancy rate (%)	47.0% (79/168)	36.9% (62/168)	0.060
Live birth rate (%)	44.0% (74/168)	35.1% (59/168)	0.094
Miscarriage rate (%)	14.0% (12/86)	25.3% (20/79)	0.065
Early miscarriage rate (%)	8.1% (7/86)	21.5% (17/79)	0.015
Late miscarriage rate (%)	5.8% (5/86)	3.8% (3/79)	0.547

Data are presented as the mean ± standard deviation and %

IVF, in vitro fertilization; PCOS, polycystic ovary syndrome; DOR, diminished ovarian reserve; FSH, follicular stimulating hormone; ET, embryo transfer

As depicted in Table 2, a binary logistic regression analysis was conducted to assess the influence of atosiban on early miscarriage rates among adenomyosis patients. Confounding factors including age, BMI, duration of infertility, previous IVF attempts, types of infertility, AMH levels, endometrial thickness, day of ET, and the proportion of transferred embryos were considered in this analysis. The multivariate analysis revealed a negative correlation between the administration of atosiban and early miscarriage rates in women with adenomyosis (adjusted OR 0.32, 95% CI 0.11–0.95, *P* = 0.040).

**Table 2:** Analyses of factors affecting early miscarriage rates in patients with adenomyosis using logistic regression

	Early miscarriage rates	
	Adjusted OR* (95% CI)	*p* value
Atosiban vs. non-atosiban	0.32(0.11–0.95)	0.040
Age (years)	1.16(0.95–1.42)	0.156
BMI (kg/m^2^)	1.00(0.89–1.14)	0.944
Infertility duration (years)	0.98(0.85–1.12)	0.731
Previous IVF attempts (n)	1.02(0.82–1.27)	0.857
Types of infertility	6.82(1.82–25.59)	0.004
AMH (ng/mL)	0.86(0.66–1.11)	0.240
Endometrial thickness (mm)	1.00(0.77–1.29)	0.967
Day of ET (%)	0.68(0.21–2.22)	0.518
Number of transferred embryos (%)	1.80(0.88–3.66)	0.107

OR, odds ratio; CI, confidence interval; BMI, body mass index; IVF, in vitro fertilization; AMH, anti-Müllerian hormone; ET, embryo transfer

*Adjustment for age, BMI, infertility duration, previous IVF attempts, types of infertility, AMH levels, endometrial thickness, day of ET, and the proportion of transferred embryos

The study population was subsequently stratified into subgroups based on age, types of infertility, and day of ET. In the subgroups stratified by age, as shown in Table 3, clinical pregnancy rates, ongoing pregnancy rates, and live birth rates were similar between the atosiban and control groups for both age groups (≥ 37 years and < 37 years). However, in the subgroup of age ≥ 37 years, the atosiban group exhibited a significantly lower rate of early miscarriage compared to the control group (9.8% vs. 33.3%, *P* = 0.005). Regarding the subgroups stratified by types of infertility, as shown in Table 4, clinical pregnancy rates, ongoing pregnancy rates, and live birth rates were not significantly different between the atosiban and control groups in either primary or secondary infertility subgroups. Nevertheless, in the subgroup of secondary infertility, the atosiban group showed a notable reduction in early miscarriage rates compared to the control group (13.1% vs. 31.1%, *P* = 0.043). For the subgroups stratified by day of ET, as shown in Table 5, the atosiban group demonstrated significantly higher live birth rates (39.6% vs. 25.2%, *P* = 0.025) and lower early miscarriage rates (4.3% vs. 31.8%, *P* = 0.001) compared to the control group for day 3 embryo transfers, but not for day 5 embryo transfers.

**Table 3:** Subgroup analysis of patients with adenomyosis stratified by age and atosiban administration

	Age < 37 years (*n* = 116)			Age ≧ 37 years (*n* = 220)		
Parameters	Atosiban (*n* = 54)	Control (*n* = 62)	*p* value	Atosiban (*n* = 114)	Control (*n* = 106)	*p* value
Age (years)	32.8 ± 2.3	34.1 ± 1.7	0.001	40.3 ± 2.3	40.6 ± 2.3	0.426
Body mass index (kg/m^2^)	23.9 ± 3.9	22.9 ± 4.4	0.198	23.6 ± 3.9	24.2 ± 4.3	0.283
Infertility duration (years)	3.1 ± 2.1	4.5 ± 2.5	0.002	5.5 ± 3.4	5.8 ± 4.1	0.487
Previous IVF attempts (n)	1.7 ± 1.1	2.3 ± 1.6	0.012	3.4 ± 2.7	3.6 ± 2.5	0.586
Primary infertility (%)	64.8%(35/54)	50.0%(31/62)	0.108	38.6%(44/114)	46.2%(49/106)	0.252
FSH (IU/L)	5.0 ± 2.4	5.2 ± 2.9	0.814	5.8 ± 3.2	5.0 ± 2.9	0.076
Anti-Müllerian hormone (ng/ml)	5.72 ± 4.35	5.11 ± 3.17	0.385	2.24 ± 2.24	2.36 ± 2.11	0.682
Endometrial thickness (mm)	11.7 ± 2.3	11.8 ± 2.3	0.869	11.6 ± 2.3	11.5 ± 2.1	0.734
ET day (%)			0.002			0.523
Day 3 ET	31.5%(17/54)	59.7%(37/62)		73.7%(84/114)	69.8%(74/106)	
Day 5 ET	68.5%(37/54)	40.3%(25/62)		26.3%(30/114)	30.2%(32/106)	
At least one top-quality embryos transferred (%)	70.4%(38/54)	80.6%(50/62)	0.197	83.3%(95/114)	77.4%(82/106)	0.264
Biochemical pregnancy rate (%)	70.4%(38/54)	56.5%(35/62)	0.122	53.5%(61/114)	43.4%(46/106)	0.134
Clinical pregnancy rate (%)	64.8%(35/54)	54.8%(34/62)	0.275	44.7%(51/114)	42.5%(45/106)	0.733
Ongoing pregnancy rate (%)	61.1%(33/54)	51.6%(32/62)	0.304	40.4%(46/114)	28.3%(30/106)	0.060
Live birth rate (%)	61.1%(33/54)	48.4%(30/62)	0.170	36.0%(41/114)	27.4%(29/106)	0.171
Miscarriage rate (%)	5.7%(2/35)	11.8%(4/34)	0.373	19.6%(10/51)	35.6%(16/45)	0.079
Early miscarriage rate (%)	5.7%(2/35)	5.9%(2/34)	0.976	9.8%(5/51)	33.3%(15/45)	0.005
Late miscarriage rate (%)	0.0%(0/35)	5.9%(2/34)	0.145	9.8%(5/51)	2.2%(1/45)	0.126

Data are presented as the mean ± standard deviation and % (n)

IVF, in vitro fertilization; FSH, follicular stimulating hormone; ET, embryo transfer

**Table 4:** Subgroup analysis of patients with adenomyosis stratified by types of infertility and atosiban administration

	Primary infertility (*n* = 159)			Secondary infertility (*n* = 177)		
Parameters	Atosiban (*n* = 79)	Control (*n* = 80)	*p* value	Atosiban (*n* = 89)	Control (*n* = 88)	*p* value
Age (years)	37.2 ± 4.6	37.6 ± 3.6	0.471	38.6 ± 3.7	38.7 ± 3.9	0.850
Body mass index (kg/m^2^)	23.8 ± 4.0	23.4 ± 4.1	0.495	23.5 ± 3.8	24.0 ± 4.5	0.490
Infertility duration (years)	4.3 ± 3.3	5.0 ± 3.3	0.193	5.1 ± 3.1	5.6 ± 4.0	0.294
Previous IVF attempts (n)	2.3 ± 2.0	3.0 ± 2.5	0.054	3.4 ± 2.7	3.3 ± 2.2	0.792
FSH (IU/L)	5.4 ± 2.8	5.1 ± 3.3	0.509	5.7 ± 3.1	5.1 ± 2.5	0.183
Anti-Müllerian hormone (ng/ml)	3.86 ± 3.96	3.18 ± 2.81	0.214	2.92 ± 2.93	3.55 ± 2.92	0.148
Endometrial thickness (mm)	11.9 ± 2.3	11.7 ± 2.4	0.487	11.4 ± 2.3	11.6 ± 2.0	0.611
ET day (%)			0.106			0.932
Day 3 ET	64.6%(51/79)	76.3%(61/80)		56.2%(50/89)	56.8%(50/88)	
Day 5 ET	35.4%(28/79)	23.8%(19/80)		43.8%(39/89)	43.2%(38/88)	
At least one top-quality embryos transferred (%)	82.3%(65/79)	80.0%(64/80)	0.714	76.4%(68/89)	77.3%(68/88)	0.891
Biochemical pregnancy rate (%)	55.7%(44/79)	43.8%(35/80)	0.132	61.8%(55/89)	51.1%(45/88)	0.153
Clinical pregnancy rate (%)	51.9%(41/79)	42.5%(34/80)	0.235	50.6%(45/89)	51.1%(45/88)	0.939
Ongoing pregnancy rate (%)	50.6%(40/79)	38.8%(31/80)	0.132	43.8%(39/89)	35.2%(31/88)	0.242
Live birth rate (%)	49.4%(39/79)	37.5%(30/80)	0.131	39.3%(35/89)	33.0%(29/88)	0.378
Miscarriage rate (%)	4.9%(2/41)	11.8%(4/34)	0.274	22.2%(10/45)	35.6%(16/45)	0.163
Early miscarriage rate (%)	2.4%(1/41)	8.8%(3/34)	0.221	13.1%(6/45)	31.1%(14/45)	0.043
Late miscarriage rate (%)	2.4%(1/41)	2.9%(1/34)	0.893	8.9%(4/45)	4.4%(2/45)	0.398

Data are presented as the mean ± standard deviation and % (n)

IVF, in vitro fertilization; FSH, follicular stimulating hormone; ET, embryo transfer

**Table 5:** Subgroup analysis of patients with adenomyosis stratified by day of embryo transfer and atosiban administration

	Day 3 embryo transfer (*n* = 212)			Day 5 embryo transfer (*n* = 124)		
Parameters	Atosiban (*n* = 101)	Control (*n* = 111)	*p* value	Atosiban (*n* = 67)	Control (*n* = 57)	*p* value
Age (years)	39.4 ± 3.6	38.5 ± 3.8	0.074	35.7 ± 4.1	37.7 ± 3.7	0.007
Body mass index (kg/m^2^)	23.5 ± 4.1	23.5 ± 4.2	0.993	23.9 ± 3.6	24.0 ± 4.6	0.866
Infertility duration (years)	5.2 ± 3.6	5.3 ± 3.5	0.914	4.0 ± 2.5	5.5 ± 4.1	0.018
Previous IVF attempts (n)	3.3 ± 2.7	3.4 ± 2.5	0.663	2.3 ± 1.9	2.6 ± 1.9	0.332
Primary infertility (%)	50.5%(51/101)	55.0%(61/111)	0.516	41.8%(28/67)	33.3%(19/57)	0.333
FSH (IU/L)	5.5 ± 2.9	5.1 ± 2.9	0.279	5.5 ± 3.0	5.1 ± 2.8	0.393
Anti-Müllerian hormone (ng/ml)	2.25 ± 2.54	2.66 ± 2.44	0.230	5.03 ± 4.00	4.77 ± 3.15	0.687
Endometrial thickness (mm)	11.4 ± 2.1	11.8 ± 2.3	0.164	12.0 ± 2.5	11.2 ± 1.8	0.035
At least one top-quality embryos transferred (%)	84.2%(85/101)	84.7%(94/111)	0.916	71.6%(48/67)	66.7%(38/57)	0.549
Biochemical pregnancy rate (%)	52.5%(53/101)	41.4%(46/111)	0.108	68.7%(46/67)	61.4%(35/57)	0.398
Clinical pregnancy rate (%)	45.5%(46/101)	39.6%(44/111)	0.385	59.7%(40/67)	61.4%(35/57)	0.847
Ongoing pregnancy rate (%)	43.6%(44/101)	27.0%(30/111)	0.012	52.2%(35/67)	56.1%(32/57)	0.664
Live birth rate (%)	39.6%(40/101)	25.2%(28/111)	0.025	50.7%(34/67)	54.4%(31/57)	0.686
Miscarriage rate (%)	13.0%(6/46)	36.4%(16/44)	0.010	15.0%(6/40)	10.0%(4/35)	0.650
Early miscarriage rate (%)	4.3%(2/46)	31.8%(14/44)	0.001	12.5%(5/40)	8.6%(3/35)	0.582
Late miscarriage rate (%)	8.7%(4/46)	4.5%(2/44)	0.430	2.5%(1/40)	2.9%(1/35)	0.924

Data are presented as the mean ± standard deviation and % (n)

IVF, in vitro fertilization; FSH, follicular stimulating hormone

The study population was subsequently categorized into subgroups based on the types of adenomyosis. Due to the small population size (*n* = 20), women with adenomyoma were excluded from the analysis. Women with diffuse adenomyosis exhibited significantly lower rates of biochemical pregnancy, clinical pregnancy, ongoing pregnancy, and live birth compared to those with focal adenomyosis (refer to supplementary Table S1). As illustrated in Table 6, the atosiban group displayed significantly higher clinical pregnancy rates (38.2% vs. 13.3%, *P* = 0.005), ongoing pregnancy rates (36.4% vs. 11.1%, *P* = 0.004), and live birth rates (32.7% vs. 11.1%, *P* = 0.011) compared to the control group for diffuse adenomyosis, but not for focal adenomyosis.

**Table 6:** Subgroup analyses (categorized by types of adenomyosis) of patients with atosiban or without

	Focal adenomyosis (*n* = 216)			Diffuse adenomyosis (*n* = 100)		
Parameters	Atosiban (*n* = 102)	Control (*n* = 114)	*p* value	Atosiban (*n* = 55)	Control (*n* = 45)	*p* value
Age (years)	37.3 ± 4.5	38.0 ± 3.7	0.213	39.0 ± 3.8	38.7 ± 3.9	0.685
Body mass index (kg/m^2^)	24.0 ± 4.0	23.5 ± 4.3	0.398	23.2 ± 3.4	23.8 ± 4.3	0.382
Infertility duration (years)	4.4 ± 3.1	5.3 ± 3.3	0.040	5.0 ± 3.5	5.4 ± 4.4	0.596
Previous IVF attempts (n)	2.7 ± 2.4	3.1 ± 2.4	0.200	3.2 ± 2.7	3.3 ± 2.2	0.952
Primary infertility (%)	49.0%(50/102)	50.9%(58/114)	0.785	45.5%(25/55)	37.8%(17/45)	0.439
FSH (IU/L)	5.7 ± 3.0	5.1 ± 3.2	0.150	4.7 ± 2.0	5.3 ± 2.1	0.181
Anti-Müllerian hormone (ng/ml)	4.03 ± 4.02	3.43 ± 2.93	0.217	2.18 ± 1.88	2.83 ± 2.08	0.104
Endometrial thickness (mm)	11.8 ± 2.2	11.8 ± 2.2	0.948	11.3 ± 2.4	11.1 ± 2.2	0.674
ET day (%)			0.162			0.562
Day 3 ET	52.0%(53/102)	61.4%(70/114)		72.7%(40/55)	77.8%(35/45)	
Day 5 ET	48.0%(49/102)	38.6%(44/114)		27.3%(15/55)	22.2%(10/45)	
At least one top-quality embryos transferred (%)	80.4%(82/102)	77.2%(88/114)	0.566	76.4%(42/55)	82.2%(37/45)	0.474
Biochemical pregnancy rate (%)	66.7%(68/102)	60.5%(69/114)	0.350	43.6%(24/55)	15.6%(7/45)	0.003
Clinical pregnancy rate (%)	57.8%(59/102)	59.6%(68/114)	0.788	38.2%(21/55)	13.3%(6/45)	0.005
Ongoing pregnancy rate (%)	52.0%(53/102)	46.5%(53/114)	0.422	36.4%(20/55)	11.1%(5/45)	0.004
Live birth rate (%)	50.0%(51/102)	43.9%(50/114)	0.367	32.7%(18/55)	11.1%(5/45)	0.011
Miscarriage rate (%)	13.6%(8/59)	26.5%(18/68)	0.072	14.3%(3/21)	16.7%(1/6)	0.885
Early miscarriage rate (%)	10.2%(6/59)	22.1%(15/68)	0.072	4.8%(1/21)	16.7%(1/6)	0.326
Late miscarriage rate (%)	3.4%(2/59)	4.4%(3/68)	0.768	9.5%(2/21)	0.0%(0/6)	0.432

Data are presented as the mean ± standard deviation and % (n)

IVF, in vitro fertilization; FSH, follicular stimulating hormone; ET, embryo transfer

## Discussion

This retrospective cohort study with PSM aimed to explore the effects of oxytocin receptor antagonists on IVF outcomes among infertile women diagnosed with adenomyosis undergoing FET HRT cycles. The study revealed that the group receiving oxytocin receptor antagonists exhibited significantly reduced rates of early miscarriage compared to the control group. Multivariate analysis demonstrated a negative association between oxytocin receptor antagonist use and early miscarriage rates in women with adenomyosis (adjusted OR 0.32, 95% CI 0.11–0.95, *P* = 0.040). Subgroup analyses stratified by age, types of infertility, and day of ET showed a significant reduction in early miscarriage rates in the subgroups of women aged ≥ 37 years, those with secondary infertility, and those undergoing day 3 embryo transfers in the atosiban group compared to the control group. Additionally, subgroup analysis based on adenomyosis types revealed significantly higher clinical pregnancy rates, ongoing pregnancy rates and live birth rates in the group receiving oxytocin receptor antagonists compared to the control group among women diagnosed with diffuse adenomyosis.

Adenomyosis, a benign gynecologic condition characterized by the presence of endometrial glands and stroma within the myometrium, has been linked to adverse reproductive outcomes [29, 31]. Numerous studies and meta-analyses have demonstrated a negative association between adenomyosis and IVF outcomes [20, 27, 28, 36, 37]. A recent systematic review and meta-analysis investigated the impact of ultrasound-diagnosed adenomyosis on IVF outcomes, revealing lower rates of live birth (OR 0.66, 95% CI 0.53–0.82), clinical pregnancy (OR 0.64, 95% CI 0.53–0.77), and higher rates of miscarriage (OR 1.81, 95% CI 1.35–2.44) among women with adenomyosis compared to those without adenomyosis [20]. Other meta-analyses have also confirmed significant reductions in rates of clinical pregnancy, ongoing pregnancy, and live birth, alongside a notable increase in miscarriage rates among infertile women with adenomyosis undergoing IVF [27, 28]. Moreover, Mavrelos et al. suggested that an increasing number of ultrasonographic features are associated with a decline in the clinical pregnancy rates in women with adenomyosis undergoing IVF-ET [38]. Additionally, Stanekova et al. reported that adenomyosis is linked to an elevated risks of miscarriage, irrespective of maternal age and BMI, following euploid blastocyst transfer [39].

The exact mechanism underlying infertility in women with adenomyosis remains poorly understood. Various mechanisms have been proposed, including anatomical distortion of the uterine cavity, dysfunctional uterine hyperperistalsis, altered steroid hormone production, elevated inflammatory mediators and oxidative stress, as well as reduced expression of implantation markers and impaired endometrial receptivity or functionality [30, 31, 40, 41]. Dysfunctional uterine hyperperistalsis, notably prevalent in women with adenomyosis, significantly influences embryo implantation [1, 25, 26]. Adenomyosis is thought to disrupt the normal architecture of the myometrium and the junctional zone, leading to aberrant uterine peristalsis, thereby adversely affecting implantation and subsequent conception. This disruption impacts the transport of sperm and embryos, as well as endometrial function and receptivity [31, 42]. Based on these findings, we hypothesized that administering an oxytocin receptor antagonist during ET could potentially improve IVF outcomes for women with adenomyosis. Our investigation indeed uncovered a positive relationship between oxytocin receptor antagonist usage and decreased rates of early miscarriage among women with adenomyosis. In a randomized controlled trial involving women with endometriosis, the endometriosis cohort showed notably higher serum oxytocin and PGF2α levels, along with increased uterine contractions compared to the tubal factor infertile cohort [43]. Furthermore, women with endometriosis demonstrated higher clinical pregnancy rates and implantation rates in the atosiban treatment group compared to the control group (58.3% vs. 38.3%, and 41.0% vs. 23.4%, respectively) [43]. Nevertheless, additional large-scale prospective studies are crucial to substantiate these findings.

Subgroup analyses, categorized by age, infertility types, and ET day, revealed a significant reduction in early miscarriage rates among women with adenomyosis following the administration of atosiban within specific subgroups: women aged ≥ 37 years, those with secondary infertility, and those undergoing day 3 embryo transfers. The uterine microenvironment in women of advanced age might be more sensitive to uterine peristalsis, potentially explaining why atosiban administration during ET yields more favorable outcomes in women with adenomyosis and advanced age. He et al. documented markedly elevated serum oxytocin and PGF2α levels, accompanied by heightened uterine contractions in the third and subsequent ET groups versus the first and second ET groups [44]. This finding suggests that patients with multiple prior unsuccessful cycles might undergo more invasive procedures, potentially enhancing the endometrial autocrine/paracrine oxytocin/oxytocin receptor system and triggering increased uterine contractions during subsequent ET attempts. Consequently, uterine peristalsis may be more frequently induced during ET in women who have previously delivered a fetus, making atosiban administration during ET potentially more beneficial for women with adenomyosis and secondary infertility. After ovulation, uterine peristalsis decreases and the uterus enters a quiescent state during the mid-luteal phase, creating an optimal environment for embryo implantation [1, 2]. Therefore, uterine peristalsis might occur more frequently during day 3 ET compared to day 5 ET. This suggests that the administration of atosiban may yield more favorable outcomes in women with adenomyosis undergoing day 3 ET. Nonetheless, the findings from the subgroup analyses should be interpreted cautiously and definitive conclusions cannot be drawn based on these data alone. Further studies are warranted to validate these observations.

In the subgroup analysis based on types of adenomyosis, adenomyosis was primarily categorized into focal and diffuse types. Han et al. demonstrated that compared to patients with focal adenomyosis or tubal infertility, those with diffuse adenomyosis experienced poorer IVF outcomes, characterized by lower clinical pregnancy and live birth rates, and higher miscarriage rates [33]. A recent systematic review and meta-analysis indicated that diffuse adenomyosis, as diagnosed by ultrasound, was associated with reduced live birth rates (OR 0.37, 95% CI 0.23–0.59) and clinical pregnancy rates (OR 0.50, 95% CI 0.34–0.75) [20]. Consistently, our study found that diffuse adenomyosis was associated with inferior IVF outcomes compared to focal adenomyosis (see supplementary Table S1). Moreover, our results indicate a favorable association between the application of an oxytocin receptor antagonist and heightened live birth rates among women with diffuse adenomyosis. Likewise, the results from the subgroup analysis should be approached with caution, as these data alone do not allow for definitive conclusions. Further larger prospective studies are necessary to confirm these results.

The current study presents several limitations that warrant consideration. Firstly, it is important to note the relatively small sample size and retrospective nature of the study, which may introduce inherent biases. Caution is advised when interpreting data from subgroup analyses due to the potential for biases stemming from the limited size of the population studied. Larger prospective studies are necessary to validate and reinforce the observed findings. Secondly, efforts were made to exclude women with sonographic evidence of endometrioma or pelvic endometriosis; however, the reliance on sonography alone may not have identified all cases accurately. Consequently, some women with both adenomyosis and endometriosis may have been inadvertently included, potentially introducing bias. Besides, embryo selection in our study relied on morphological grading rather than euploidy assessment, as PGT-A is not commonly utilized in our center. Therefore, the possibility of confounding effects from embryo aneuploidy should be considered. Moreover, the decision to prescribe an oxytocin receptor antagonist was influenced by patient preferences and individual considerations after extensive consultations with a physician, which may have introduced bias into the study.

In conclusion, the administration of an oxytocin receptor antagonist during FET may potentially decrease the early miscarriage rates in women with adenomyosis.

### Electronic supplementary material

Below is the link to the electronic supplementary material.

Supplementary Material 1

Supplementary Material 2

## Data Availability

Data is provided within a Additional file 2.

## Abbreviations

AMH: anti-Mullerian hormone
BMI: body mass index
CI: confidence interval
ET: embryo transfer
FET: frozen embryo transfer
FSH: follicular stimulating hormone
GnRH: gonadotropin-releasing hormone
HRT: hormone replacement therapy
IVF: in vitro fertilization
MUSA: morphological uterus sonographic assessment
OR: Odds ratio
PGT-A: preimplantation genetic testing cycles for aneuploidy
PSM: propensity score matching
