# Supplementary material for: Improvement of early miscarriage rates in women with adenomyosis via oxytocin receptor antagonist during frozen embryo transfer-a propensity score-matched study
Source: Reprod Biol Endocrinol. 2024 Jul 12;22:79. doi: 10.1186/s12958-024-01255-1 (PMC11241821; doi:10.1186/s12958-024-01255-1)
Supplement: Supplementary file 1 — Supplementary Material 1 [file 12958_2024_1255_MOESM1_ESM.docx]

**Supplementary Table 1 Baseline and cycle characteristics of patients with focal or diffuse adenomyosis undergoing frozen embryo transfer**

| Parameters | Focal adenomyosis  (n=216) | Diffuse adenomyosis  (n=100) | *p* value |
| --- | --- | --- | --- |
| Age (years) | 37.7±4.1 | 38.9±3.8 | 0.013 |
| Body mass index (kg/m^2^) | 23.8±4.2 | 23.5±3.8 | 0.568 |
| Infertility duration (years) | 4.9±3.2 | 5.2±3.9 | 0.516 |
| Previous IVF attempts (n) | 2.9±2.4 | 3.3±2.5 | 0.282 |
| Types of infertility (%) |  |  | 0.185 |
| Primary infertility | 50.0%(108/216) | 42.0%(42/100) |  |
| Secondary infertility | 50.0%(108/216) | 58.0%(58/100) |  |
| FSH (mIU/mL) | 5.4±3.1 | 5.0±2.1 | 0.169 |
| Anti-Müllerian hormone (ng/mL) | 3.71±3.49 | 2.48±1.99 | <0.001 |
| Endometrial thickness (mm) | 11.8±2.2 | 11.2±2.3 | 0.027 |
| ET day (%) |  |  | 0.002 |
| Day 3 ET | 56.9% (123/216) | 75.0% (75/100) |  |
| Day 5 ET | 43.1% (93/216) | 25.0% (25/100) |  |
| At least one top-quality embryos transferred (%) | 78.7% (170/216) | 79.0% (79/100) | 0.952 |
| Biochemical pregnancy rate (%) | 63.4% (137/216) | 31.0% (31/100) | <0.001 |
| Clinical pregnancy rate (%) | 58.8% (127/216) | 27.0% (27/100) | <0.001 |
| Ongoing pregnancy rate (%) | 49.1% (106/216) | 25.0% (25/100) | <0.001 |
| Live birth rate (%) | 46.8% (101/216) | 23.0% (23/100) | <0.001 |
| Miscarriage rate (%) | 20.5% (26/127) | 14.8% (4/27) | 0.500 |
| Early miscarriage rate (%) | 16.5% (21/127) | 7.4% (2/27) | 0.227 |
| Late miscarriage rate (%) | 3.9% (5/127) | 7.4% (2/27) | 0.432 |

Data are presented as the mean ± standard deviation and %.

IVF, in vitro fertilization; FSH, follicular stimulating hormone; ET, embryo transfer
